# Supplementary material for: Participatory Co-Design and Evaluation of a Novel Approach to Generative AI-Integrated Coursework Assessment in Higher Education
Source: Behav Sci (Basel). 2025 Jun 12;15(6):808. doi: 10.3390/bs15060808 (PMC12189063; doi:10.3390/bs15060808)
Supplement: Supplementary file 1 [file behavsci-15-00808-s001.zip › behavsci-3647282-supplementary.pdf]

Participatory co-design and evaluation of a novel approach to generative AI-integrated coursework assessment in higher education

Alex F. Martin [1,*], Svitlana Tubaltseva [2], Anja Harrison [1] and G. James Rubin [1]

[1] Department of Psychological Medicine, Institute of Psychiatry, Psychology and Neuroscience, King's College London, London WC2R 2LS, UK; anja.harrison@kcl.ac.uk (A.H.); gideon.rubin@kcl.ac.uk (G.J.R.)

[2] School of Liberal Arts, Richmond American University London, London W4 5AN, UK; tubalts@richmond.ac.uk

* Correspondence: alex.f.martin@kcl.ac.uk

Supplementary materials

Supplement A: GRIPP2-SF reporting checklist

Guidance for Reporting Involvement of Patients and the Public short form (GRIPP2-SF) checklist.

The *Guidance for Reporting Involvement of Patients and the Public* (GRIPP) was developed to address inconsistent reporting practices in patient and public involvement (PPI). It supports researchers, patients, carers, and members of the public in enhancing the quality, consistency, and transparency of PPI reporting, thereby strengthening the evidence base for PPI in research (Staniszewska et al., 2017).

The GRIPP checklist is provided below.

Table S1. GRIPP2 short form

Section and topic	Item	Reported on page number
1: Aim	Report the aim of PPI in the study	3, 4
2: Methods	Provide a clear description of the methods used for PPI in the study	4-8
3: Study results	Outcomes - Report the results of PPI in the study, including both positive and negative outcomes	8-15
4: Discussion and conclusions	Outcomes - Comment on the extent to which PPI influenced the study overall. Describe positive and negative effects	16-17
5: Reflections / critical perspective	Comment critically on the study, reflecting on the things that went well and those that did not, so others can learn from this experience	16-17

PPI = Patient and public involvement

Supplement B: Focus group and interview schedules

Indicative discussion schedule for the focus groups (student participants) and interviews (teaching staff participants).

Focus group schedule (students)
Section 1: Putting the participants at ease

Introductions

1. Do you live in London or elsewhere?
2. What brought you to the MSc and what have you enjoyed the most?

Section 2: Establishing knowledge and experience

1. What is generative AI?
2. How do you think AI is being used by students in their studies?
3. What do you consider the benefits of AI to your studies?
4. Do you have any concerns about AI being used in academic study?

Prompt: or research more broadly?

Section 3: Experience of the mock assessment

1. How did you find the AI workshop?
   Prompt: was it useful for completing the coursework / was anything missing that would have helped you complete the coursework?
2. **Feasibility**
   a. How much time did it take you to complete the coursework compared to traditional methods?
   b. Was the coursework brief clear and easy to follow?
   c. Honest blog: How did you find the process of using AI tools to assist in writing the blog?
   Cheat blog: How did you find the process of using AI tools to write the blog?
   Prompt: What tools did you use / What did you do / Did you encounter any challenges when using the AI tools?
3. **Acceptability**
   a. What was the purpose of the assessment?
   b. Did you feel you attained the learning outcome?
   c. What are your thoughts on the quality of the AI-generated content?
   d. How do you feel about integrating AI tools into the assessment process?
   Prompt: Would you support the adoption of AI-assisted assessment in other modules?
   e. Do you believe this new approach fairly evaluates your abilities?
4. **Integrity and implementation**
   a. Honest blog only: Did you feel tempted to rely solely on AI for the assignment? Why or why not?
   b. Cheat blog only: Did ‘cheating’ on the blog affect your attainment of the learning objectives for the assignment?
   c. What kind of guidance do you think is necessary for students to use AI tools ethically in assessments?

Interview schedule (teaching staff)
Section 1: Putting the participants at ease

1. Which module do you teach on, have you been teaching there for a while?

Section 2: Establishing knowledge and experience

1. What is generative AI?
2. Before taking part in this study, have you used generative AI for your teaching or research?
3. Are you aware of students using AI, what do they use it for?

4. What do you consider the benefits of AI for teaching?

Prompt: What do you consider the benefits of AI for students?

5. Do you have any concerns about AI being used in teaching?

Section 3: Experience of the mock coursework

1. Did you attend the AI workshop, if so, how did you find it?

   Prompts: was it useful for marking the coursework / was anything missing that would have helped you mark the coursework?

2. **Feasibility**

   a. How much time did it take you to complete the marking compared to traditional methods?

   b. Was the coursework brief clear and easy to follow?

   c. How did you find marking using the AI rubric?

   d. How did you find the process of marking the mock assessment?

   Prompt: Did you encounter any challenges when marking?

   Prompt: what else do you need?

3. **Acceptability**

   a. What are your thoughts on the quality of the AI-generated content?

   b. How do you feel about integrating AI tools into the assessment process?

   Prompt: Would you support the adoption of AI-assisted assessment in other modules?

   c. Do you believe this new approach fairly evaluates students' abilities?

4. **Integrity and implementation**

   a. Were you able to distinguish between the 'honest' and 'cheat' blogs?

   b. What kind of guidance do you think is necessary for students to use AI tools ethically in assessments?

   c. How can we ensure that the assessment remains a true test of a student's own work and critical thinking?

Supplement C: Coproduced assessment materials

The final coproduced assessment materials, including the workshop proformas, assessment brief, and marking matrix.

These documents remain in draft, pending pilot testing during the upcoming module rollout.

1. Workshop proformas (inc a session for staff??) – TBC with Lana
2. Assessment brief
    a. ChatGPT recommendations for improving readability and accessibility
3. Marking matrix

1. Workshop proformas

AI Workshop Foundation 1

Pre-session reading: Cheng, A., Calhoun, A., & Reedy, G. (2025). Artificial intelligence-assisted academic writing: recommendations for ethical use. *Advances in Simulation*, *10*(1), 22. https://doi.org/10.1186/s41077-025-00350-6

Duration: 1-2 hours

Learning outcomes

By the end of this session, students will be able to:

1. Explain key ethical considerations related to the use of generative AI in academic work.
2. Apply prompt engineering strategies (e.g. persona pattern, flipped prompting) to explore diverse perspectives on a topic.
3. Evaluate AI-generated outputs for accuracy, bias, and representation.
4. Reflect critically on the ethical implications of using AI tools in the assessment context.

Session purpose

In this session, you will explore how generative AI can be used in academic work and reflect on the ethical considerations that come with it. You'll learn how to write effective prompts, identify common issues with AI outputs, and begin thinking critically about how these tools can support and challenge your learning.

Warm up

1. Elicit from participants what they know about generative AI tools.

Ethics and AI

2. Idea generation activity on the ethical use of AI, tailored to the specific context of the assessment. Participants are split into small groups. At the end of discussion, each group gives a mini presentation.

   Instructor's feedback

3. The instructor presents the key points of the ethical use of AI in academic work:
   a. Undeclared and/or unauthorised use of AI tools can lead to academic misconduct
   b. Fabrication and falsification of data because of AI hallucinations
   c. Bias and lack of diversity
   d. Issue of copyright
4. Revision of key strength and weaknesses of AI in academic work.

Prompt writing. Input section:

5. The instructor shows examples of how efficiently to use AI features. Focus on good/bad prompt writing. Introduction of Persona pattern and Flipped prompting.

Practice section:

6. Participants are given a generic topic (relevant to their programme and which they know well) where they choose their context. They are encouraged to use Flipped prompting to gain a summary of different angles of the topic with the specific focus on the diversity of sources and information cited in the input.

Once the information is collected, participants are asked to use a Persona pattern prompting to generate a blog based on the AI input they have got.

Critical evaluation and reflection

7. Participants are divided into groups and critically reflect on their experience using different prompts to generate the information. They evaluate their blogs in terms of the ethics and diversity.
    a. What sources have been cited for the blog?
    b. Are their any references to non-white, non-Western countries, examples, people?
    c. Can you detect any falsification or bias in the final blog?

Wrapping up the workshop

8. Group reflections to consolidate learning
    a. What did you find most surprising or challenging about how the AI responded to your prompts?
    b. How do your views about using AI in academic work compare to your views before the session?
    c. What ethical considerations feel most relevant to your own use of AI in future assessments?

Optional homework: choose another topic and use different types of prompts to create a blog. Analyse it according to the ethics and representativeness of views there.

AI Workshop Foundation 2

Duration: 1-2 hours

Learning outcomes

By the end of this session, students will be able to:

1. Use generative AI tools to draft a structured blog based on an academic article, aligned with the assessment brief.
2. Edit and refine AI-generated content to meet academic and professional standards (e.g. tone, referencing, accessibility).
3. Critically appraise the strengths and limitations of AI-assisted academic writing using the assessment rubric.
4. Develop an action plan for completing the assessment, specifying appropriate and ethical use of AI tools.

Session purpose

In this session, you'll apply what you've learned to a real academic task. You'll use AI tools to help you draft and refine a blog, like the one you'll complete for your assessment. You'll also evaluate the strengths and limitations of the AI-generated content and develop a plan for how you'll use AI ethically and effectively in your coursework.

Warm up

1. Revision of the previous workshop. Students are split into two groups and discuss their homework and what they have learnt from it.

Practice section (creating a blog)

2. Revision of the assignment brief (blog writing) and learning outcomes.
3. Participants read an article and are instructed to create a blog using a generative AI tool of their choice that meets the assessment brief (i.e. free to use and clearly liked to a LLM such as CoPilot or ChatGPT). They receive a step-by-step demonstration of how to generate AI-produced blog (see below).

Make sure that your blog consists of the following sections:

- *Introduction/background (a brief introduction to the subject area)*
- *Methods*
- *Result*
- *Conclusion (a summary of the research conclusions)*
- *Strengths and limitations*
- *Implications for practice*

The objectives are to encourage participants to experiment with AI, learn from their personal experiences and the input section earlier, and enhance their critical awareness of the pros and cons of AI for blog creation.

4. Editing stage of the blog (structure, stance, proof reading). Students follow clear requirements (see below).
    - *Put all sections together according to the required structure and ask AI to add an introduction and conclusion to the blog*
    - *Ask AI to add citations and create a reference list following APA Referencing style*
    - *Ask AI to proofread and fact check the draft*
    - *Ask AI to change the register of the draft, aim it at health, mental health and social care professionals and keep things jargon-free and as accessible as possible.*
    - *Ask AI to create a title of the blog (titles should start with the main keyword or phrase from your blog, and should try to include the patient/problem and intervention covered by the study)*

Critical analysis

5. Participants critically appraise and analyse the final draft according to the grading criteria for this assignment. Participants complete the table.

Assignment criteria	Pros of using AI tool	Cons of using AI tool
Interpretation of academic papers: identification of key points		
Accuracy of the produced content		
Relevance and accuracy of the added citations and references		
Critical thinking		
Creative and innovative thinking		
Academic literacy: register, tone, referencing style		

6. Group discussion of strength and weaknesses of generative AI tools for this academic assignment.

Revision and action plan

7. Participants are asked to create an action plan they will use to complete the real assignment, including what they will or will not use AI for.

Wrapping up the workshop

8. Group reflections to consolidate learning
    a. How did the AI-generated blog compare to what you would have written on your own?
    b. What decisions did you make when editing or combining AI outputs, and why?
    c. How do you plan to use AI in your assessment, and what steps will you take to ensure it reflects your own critical thinking?
9. Feedback questions
    a. Have you found the workshop helpful?
    b. What have you learnt from the workshop?
    c. What would you like to add to the workshop in the future?

ChatGPT recommendations to improve the workshop proformas:

🔧
Suggestions for Improvement
1.
Clarify Workshop Objectives at the Start
Add a brief statement at the beginning of each workshop outlining its purpose and how it supports the assessment. E.g.:
“This session is designed to help you explore how generative AI tools can support and challenge your academic writing, while equipping you to use them ethically and effectively in your upcoming assessment.”
2.
Reinforce Alignment With Assessment Brief
Explicitly mention at the start of Workshop 2 that the session mirrors the actual assessment process, including tasks students will be required to complete.
3.
Use Reflective Prompts
Consider including a short reflective activity or prompts after each session to consolidate learning. E.g.:

- *“What surprised you most about the AI-generated content?”*
- *“How might you use AI differently in your real assessment based on today’s experience?*

4.
Encourage Pre-Workshop Reading
For Workshop 2, students could be sent the academic article in advance to maximise hands-on time during the session.
5.
Incorporate Student Examples (if available)
In Workshop 2, if anonymised student work is available from earlier pilots, showing a real example of a blog and commentary could enhance clarity and engagement.

2. **Assessment brief and ChatGPT recommendations for improving readability and accessibility.**

Assessment brief: write a blog using generative AI

1. Introduction

This coursework offers you the opportunity to read, appraise and communicate the findings of high-quality research in the form of a blog. You will use generative AI to help you produce the content and critically analyse the application and output of the AI tools.

This assessment is designed to support the development of two interrelated skill sets: critical appraisal and AI literacy. As generative AI becomes more integrated into professional and academic settings, students must not only understand how to use these tools effectively but also how to critically evaluate their outputs. This exercise promotes ethical awareness, digital fluency, and the ability to distinguish human insight from machine-generated content. These are essential skills for future researchers, clinicians, and practitioners.

Why blogs?

Before you read the instructions on how to plan and write your blog, it might be helpful to understand why we use blogs in our assessment.

The blog format supports key academic and professional skills:

- Engagement with current research and formation of independent opinions
- Effective communication to diverse audiences - essential for roles in academia, mental health, policy, and beyond
- Creativity and innovation, with structured support to develop these competencies during your course

Why generative AI?

Generative AI is increasingly used across sectors, including academic research and mental health practice. It offers powerful tools for supporting writing, summarising complex material, and generating ideas. However, its use also requires critical thinking and ethical awareness.

Opportunities:

- Enhances idea generation and writing efficiency
- Supports content refinement and academic expression
- Prepares students for AI-integrated workplaces

Risks and Ethical Considerations:

- Academic integrity: Risk of over-reliance impacting learning or unacknowledged AI input
- Bias and accuracy: AI may hallucinate facts or reflect biased training data
- Environmental impact: Large-scale AI tools require significant computational power, contributing to energy use and carbon emissions
- Transparency: Students must understand how and when to disclose AI use

Use of generative AI

You are required to use two AI tools for writing and editing your blog.

To support this, you will attend a workshop titled *Using AI in Coursework*, which will cover:

- Types of generative AI relevant to the assessment
- How to write effective prompts
- Common pitfalls in using AI tools
- Ethical considerations in academic contexts

2. Instructions for the mock blog coursework

In this coursework, you will use generative AI tools to create a blog from an academic paper.

1. Choose an empirical paper from the two provided below.
2. Critically appraise the empirical paper. *You may wish to use one of the suggested tools from the Tips section below.* ***You do not need to submit your own critical appraisal, but this step is essential to understand how successful AI has been at summarising the empirical paper***.
3. Use two different generative AI tools to generate a blog based on the paper, that meets the blog guidance. These must be tools that are free to use and access ChatGPT-4 or an equivalent model. State the tools that you have used ad provide a link to them in the appendix and the reflective account (see below).
4. Identify the strengths and weaknesses of the two AI-generated blogs and then produce your own final version of the blog, which includes the best elements of the two blogs and any revisions or original text that you believe should be included. *You may wish to use one of the suggested reflection frameworks from the Tips section below.*
5. The coursework that you submit to us should be a maximum of 2,000 words, plus appendices which are not included in the word count.
6. The coursework must include the following, in this order:
    1. The final blog (1,000 words – 50% of total word count).
    2. Commentary (1,000 words – 50% of total word count), including:
        - 2.1 A critique of the two original AI-generated blogs. This should include any errors produced by AI, for example, inaccurate reporting or misinterpretation of the empirical study findings. The commentary should state which AI tools you used.
        - 2.2 Reflections of how you created your final blog and rationale for revisions.
    3. In the appendix (no word limit):
        - 1.1 The names of the free generative AI tools that you used, including the LLM model they access, and provide links to both.
        - 1.2 The full, unedited prompts you used to generate the AI blogs.
        - 1.3 The final blog, with all new content clearly marked. Highlight any sentences you have added or amended. Leave all AI-generated text unhighlighted to clearly distinguish your contributions.
        - 1.4 The original versions of the blogs produced by the AI tools. There is no need to revise or polish these versions, we are interested in the original outputs

You can choose to create the blogs from EITHER of the below (not both):

Paper 1

Paper 2

Teaching team note: papers should be selected that are complex articles with intentional limitations or errors that are not highlighted by the study authors.

3. Learning outcomes

1. **Critical appraisal**: Students will demonstrate the ability to critically appraise academic content by:
   i. Evaluating an empirical research article using an established checklist (e.g. CASP)
   ii. Assessing the accuracy, relevance, and limitations of AI-generated content in relation to the original empirical paper
   iii. Comparing outputs from different AI tools, identifying their strengths and weaknesses in academic content generation
2. **Generative AI literacy**: Students will develop foundational AI literacy by using generative tools to support academic writing. They will demonstrate an understanding of AI capabilities and limitations, including the ability to identify common errors such as fabrication or hallucination.
3. **Editorial and reflective judgment**: Students will apply editorial judgment to revise AI-generated content, integrating critical analysis and original insight. They will reflect on their use of AI tools and articulate the rationale for content modifications in alignment with accuracy, academic standards, and ethical considerations.

4. How Your Work Will Be Assessed

Your final submission will be marked using an adapted version of the standard programme-wide marking criteria. In addition to evaluating your understanding and critical appraisal of the academic article, markers will also assess how you:

- Evaluate the strengths and limitations of the AI-generated content
- Apply editorial judgement to revise and integrate content from multiple sources (AI and original)
- Reflect critically on your own use of AI tools, including prompt design and ethical considerations

The marking matrix assesses both your academic skills and your ability to engage meaningfully with AI as a tool. You will not be judged on how well the AI performed, but rather on how well you critically used and responded to its outputs.

5. Tips

1. Before you start using generative AI, ensure that you have thoroughly read and understood the paper. To assist with this, you must use a critical appraisal checklist, such as those available at https://casp-uk.net/casp-tools-checklists/. Alternatively, The Collaborative Library has checklists for most types of study in more accessible language: https://thecollaborativelibrary.com/glossary-of-terms-and-jargon-buster/.
2. You **do not need to submit your critical appraisal as part of your assessment**, but you must complete this step to verify that AI has correctly interpreted the paper in your blog.
3. When crafting prompts, be specific, concise, and test variations. Iterative refinement often improves AI responses.
4. When evaluating the AI output, keep the following points in mind:
   - AI can sometimes produce inaccurate information, known as 'hallucinations'; do not assume that everything it writes is correct
   - AI will only summarise the content provided by the authors of the paper. Do not assume that the authors' work is free from errors

5. Your blog should clearly reflect your own writing style. Avoid making it sound like it was written by AI. This can happen if the tone of the writing is too formal or if the blog only includes the limitations and implications suggested by the authors. Make sure your final blog incorporates your unique voice and original perspectives.
6. To help you write the reflective account in 2.2 in the Instructions section above, you may wish to use a reflective framework. We recommend Kolb's Experiential Learning Cycle for this activity: https://www.simplypsychology.org/learning-kolb.html

6. Blog guidance

Your final blog should be 1,000 words, excluding the links and references presented at the end. You should write in clear, concise and informal language, using your own natural voice.

Your blog should be aimed at health, mental health and social care professionals, so you don't need to write in lay friendly language for patients. However, research shows that professionals appreciate simple and clear language too, so try to keep things jargon-free and as accessible as possible.

The aim of your blog is to help your target audience to engage with the latest research. Your role is to:

1. Summarise the new piece of evidence
2. Highlight the strengths and limitations of the research
3. Provide your own commentary on the evidence (how should this impact on practice, policy and future research)
4. Provide a personal perspective, if possible (your own lived experience, your clinical perspective, your experience as a care-giver etc.)

Title

- The shorter the better!
- Titles should start with the main keyword or phrase from your blog, and should try to include the patient/problem and intervention covered by the study, e.g.
    - Antidepressants for depression in pregnancy
    - Behavioural activation not inferior to CBT for depression
- If you wish, you can include a second part to the title using a colon, e.g.
    - Antidepressants for depression in pregnancy: new systematic review suggests they are safe and effective
    - Behavioural activation not inferior to CBT for depression: the COBRA RCT

Background: approximately 150 words

- A brief introduction to the subject area
- Setting the scene
- Presenting background statistics (e.g. incidence, prevalence)
- Citing other related research
- Introducing the new evidence that is the focus of your blog
- The introduction needs to capture the attention of the reader and make them want to read on, so consider using jokes, anecdotes or terrible elf puns at the beginning to entertaining people and draw them in.

Methods approximately 150 words

- A brief summary of the methodology of the new evidence
- Readers will focus on this section if they are evaluating the robustness of this new evidence. It's therefore important that you focus on the aspects of the methodology tackled by the critical appraisal checklists.
- Results: approximately 200 words
- A succinct presentation of the research results
- Statistical results can be included.
- Complicated data can be tabulated if necessary.

Conclusions: approximately 100 words

- A summary of the research conclusions
- Can include a quote from the study if appropriate, e.g. The authors concluded:

Strengths and limitations: approximately 200 words

- This is where you present your critical appraisal of the evidence. It's the most important section in your blog.
- This should include your view on the methodological weaknesses of the study.
- It should not simply be a repetition of the limitations highlighted by the researchers themselves in their paper.
- Remember, be confident and critical.

Implications for practice: approximately 200 words

- The final section is where you should relate the evidence to practice.
- Should this new evidence have an impact on practice?
- What changes should be made to policy as a result of these new findings?
- What new research avenues does this evidence open up?
- You can also use this closing section to share your own personal perspective on the topic (your own lived experience, your clinical perspective, your experience as a care-giver etc). The most powerful blogs are those that provide this personal view, relating the new evidence to a human story.

Conflicts of interest

- If you have any conflicts of interest, please state these at the end of your blog. Potential conflicts include:
  - Having personal involvement in the study
  - Knowing the researchers involved in the study and working with them on other projects
  - Being involved in the reviewing or publication of the study
  - Working on a similar piece of research yourself
  - Paid employment or self-employment
  - Grant funding
  - Voluntary appointments
  - Memberships
  - Decision-making positions
  - Other financial interests

References and links

- A complete bibliography of all the evidence cited in the blog
- Starting with the reference for the actual piece of new evidence you are highlighting (Primary paper) and continuing with a list of all the other references (Other references)
- Please add links to the full-text or abstracts of your references if they are available online.
- References should be cited and formatted using a consistent referencing style, for example, APA 7.
- Papers with full text freely available should be linked to like this:
  - Jones M, Smith P, Anderson T. et al (2014) Antidepressants are safe and effective for women with depression who are pregnant (PDF). BMJ 2014 33(6) 172-183
  - Link to the full-text HTML if it's available. If not, link to the PDF with (PDF) in the link text
- Papers with only abstracts freely available should be linked to like this:
  - Jones M, Smith P, Anderson T. et al (2014) Antidepressants are safe and effective for women with depression who are pregnant. BMJ 2014 33(6) 172-183. [PubMed abstract]

Deadline

Submit your blog by **date and time**

ChatGPT recommendations to improve the assessment brief:

✅ Strengths

- Clear breakdown of sections with approximate word counts
- Strong rationale for using blogs and AI
- Inclusion of ethical and environmental considerations
- Explicit learning outcomes aligned with assessment tasks
- Helpful “Tips” section and external resources

🔧 Suggested Improvements

1. Clarify AI Tool Access and Equality

- Current: “Use two different generative AI tools (Copilot using the ‘Precise’ and ‘Creative’ conversation styles...)”
- Suggestion: Explicitly state that both tools are free and equally accessible to all students to avoid equity concerns

E.g. “Use two different generative AI tools — Copilot in ‘Precise’ and ‘Creative’ modes — both of which are freely available and provide access to ChatGPT-4.”

2. Refine Instructions for Submission Structure

- Consider bullet-pointing the required components under “The coursework must include the following...” for quicker comprehension.

Suggested format:

- Final blog (1,000 words)
- Commentary (1,000 words), including:
- A critique of the two original AI-generated blogs
- Reflections on how you created your final blog and rationale for revisions
- Appendices (not included in the word count):
- Full, unedited prompts
- Original AI-generated blogs

3. Clarify Assessment Purpose

- Add one to two sentences early in the document linking the blog and AI use explicitly to assessment integrity and skill development. This will reinforce the rationale behind the exercise.

E.g. “This assessment is designed not only to develop your academic writing and communication skills, but also to build your ability to critically appraise and ethically engage with generative AI tools — skills increasingly relevant across sectors.”

4. Streamline “Why Generative AI?”

- Consider consolidating the ethical and environmental points into a new subheading: “Risks and Ethical Considerations,” so the section doesn’t become too dense.

5. Use Consistent Tone in “Tips” Section

- Some tone is slightly informal (e.g., “don’t worry about ‘tidying’ them”). Maintain a friendly tone but consider aligning more closely with the formal tone used elsewhere.

Replace: “don’t worry about ‘tidying’ them...”

With: “There is no need to revise or polish these versions — we are interested in the original outputs.”

6. Add Brief Note on AI Prompting Best Practices

- A short addition in the Tips section could guide students on refining prompts:

E.g. “When crafting prompts, be specific, concise, and test variations. Iterative refinement often improves AI responses.”

7. Strengthen Link Between Blog and Assessment Criteria

- Consider referencing how the blog will be evaluated (e.g., clarity, critical analysis, originality, etc.) or mention that this is detailed in the marking matrix.

3. Marking matrix

MSc PROGRAMME

Marking matrix: write a blog using generative AI

The coursework must include the following, in this order:

4. The final blog (1,000 words – 50% of total word count).
5. Commentary (1,000 words – 50% of total word count), including:
   - 2.3 A critique of the two original AI-generated blogs. This should include any errors produced by AI, for example, inaccurate reporting or misinterpretation of the empirical study findings. The commentary should state which AI tools you used.
   - 2.4 Reflections of how you created your final blog and rationale for revisions.
6. In the appendix (no word limit):
   - 3.1 The names of the free generative AI tools that you used, including the LLM model they access, and provide links to both.
   - 3.2 The full, unedited prompts you used to generate the AI blogs.
   - 3.3 The final blog, with all new content clearly marked. Highlight any sentences you have added or amended. Leave all AI-generated text unhighlighted to clearly distinguish your contributions.
   - 3.4 The original versions of the blogs produced by the AI tools. There is no need to revise or polish these versions, we are interested in the original outputs

Here are the learning outcomes:

1. **Critical appraisal**: Students will demonstrate the ability to critically appraise academic content by:
   - i. Evaluating an empirical research article using an established checklist (e.g. CASP)
   - ii. Assessing the accuracy, relevance, and limitations of AI-generated content in relation to the original empirical paper
   - iii. Comparing outputs from different AI tools, identifying their strengths and weaknesses in academic content generation
2. **Generative AI literacy**: Students will develop foundational AI literacy by using generative tools to support academic writing. They will demonstrate an understanding of AI capabilities and limitations, including the ability to identify common errors such as fabrication or hallucination.
3. **Editorial and reflective judgment**: Students will apply editorial judgment to revise AI-generated content, integrating critical analysis and original insight. They will reflect on their use of AI tools and articulate the rationale for content modifications in alignment with accuracy, academic standards, and ethical considerations.

Note: This is an authentic assessment task. Selected blog submissions may be considered for publication, subject to editorial review.

**CHECKLIST FOR EXAMINERS for part 1: the final blog (1000 words)**	
**The blog**	• Students are asked to generate two blogs using different generative AI tools, and then produce their own final version of the blog. The final version should include the best elements of the two blogs.<br>• This final blog should be assessed using the following criteria referencing the taught postgraduate generic marking criteria (below).

**Title**	• The shorter the better!<br>• Titles should start with the main keyword or phrase from the blog and should try to include the problem and intervention covered by the study.<br>• May include a second part to the title using a colon.
**Background**<br>Approx 150 words	• A brief introduction to the subject area.<br>• Highlighting the importance of the topic (e.g. incidence, prevalence of studied condition).<br>• Citing other related research and/or blogs.<br>• Introducing the new evidence that is the focus of the blog.<br>• Written in clear, concise and informal language, using a natural human voice.<br>• Aimed at health, mental health and social care professionals, who may or may not be experts on the subject area.
**Methods**<br>Approx 150 words	• Blog readers focus on this section if they are evaluating the robustness of the new evidence.<br>• May focus on aspects of methodology tackled by the CASP critical appraisal checklists: https://casp-uk.net/casp-tools-checklists/.<br>• This section needs to be concise and cannot be fully comprehensive.
**Results**<br>Approx 200 words	• A succinct presentation of the research results.<br>• Statistical results can be included.<br>• Complicated data can be tabulated if necessary.
**Conclusions**<br>Approx 100 words	• A summary of the research conclusions.<br>• Can include a quote from the study if appropriate, e.g. The authors concluded*: ''CBT for psychosis significantly improves psychotic symptoms''*.
**Strengths and limitations**<br>Approx 200 words	• Critical appraisal of the evidence. It's the most important section in the blog.<br>• This should include the methodological weaknesses of the study.<br>• It should not simply be a repetition of the limitations highlighted by the researchers themselves in their paper.<br>• This section should demonstrate the student's confidence and critical appraisal skills.
**Implications for practice**<br>Approx 200 words	• The final section is where evidence is related to practice.<br>• Should the new evidence have an impact on practice?<br>• What changes should be made to policy because of the new findings?<br>• What new research avenues does this evidence open?<br>• This closing section can be used to share a personal perspective on the topic (lived experience, clinical perspective, experience as a caregiver etc). The most powerful blogs are those that provide this personal view, relating the new evidence to a human story.
**Conflicts of interest**	• These should be stated at the end of the blog.
**References and links**	• A complete bibliography of all the evidence cited in the blog, starting with the reference for the actual piece of new evidence (Primary paper) and continuing with a list of all the other references (Other references).<br>• References should be cited and formatted using a consistent referencing style, for example, APA 7.<br>• If freely available online, the full-text or abstracts of all references should be linked (see full instructions in the document 'Instructions for Blog Coursework').

**CHECKLIST FOR EXAMINERS for part 2: commentary (1000 words)**

**AI tools**	• Students should use two different generative AI tools that are freely available and accessible.<br>• These must be stated in the commentary alongside the LLM that the model is accessing.<br>• For example, Microsoft Copilot (GPT-4) or Poe (Claude).
**Critical integration**	• A critique of the two, original AI created blogs. This should include any errors produced by AI, for example, inaccurate reporting or misinterpretation of the empirical study findings.<br>• A reflective account of how the student created their own final version and why they made any revisions or other decisions.
**Appendix**	• The full, unedited prompts used to generate the AI blogs.<br>• The original versions of the blogs produced by the AI tools.

A key element of this exercise is demonstrating that students can apply appropriate critical appraisal skills to the original paper, and to assess whether the AI tools have correctly interpreted the paper.

To reflect this in your marking, please include the guidance below alongside those provided in the Taught Postgraduate Generic Marking Criteria.

1. Critical understanding and application of generative AI

- **Distinction (≥70) Demonstrates deep insight into AI tool usage and revision choices, with critical and ethical reflection.**

  Skilful integration of generative AI outputs with clear human oversight. Evidence of refinement and personalization beyond the AI-generated draft. Demonstrates critical and ethical use of AI, including a robust reflection on the limitations and biases of AI-generated content. Does not include any errors from the AI generated content in the final blog and includes a thoughtful discussion of any errors AI created.

- **Merit (60-69) Offers thoughtful commentary with some depth of analysis on AI outputs and revision process.**

  Effective use of generative AI with some evidence of human refinement. Good understanding of ethical AI use with some reflection on limitations and biases. Does not include any errors from the AI generated content in the final blog and includes some discussion of any errors AI created.

- **Pass (50-59) Basic explanation of AI use and revision, with limited critical engagement.**

  Basic use of AI-generated content with minimal refinement. Sound understanding of ethical considerations with limited reflection. Does not include any errors from the AI generated content in the final blog.

- **Fail (<50)**

  Over-reliance on AI without sufficient human oversight. Lacks ethical considerations or reflection on AI use. Includes errors from the AI generated content in the final blog, for example, inaccurate statement of results or limitations.

ChatGPT recommendations to improve the assessment brief:

✅
Strengths

- Clear two-part structure (Blog + Commentary).

- Integration of AI-specific assessment criteria alongside KCL's generic PG marking framework.
- Learning outcomes are well-matched to rubric content.
- Detailed guidance on each blog section improves transparency for students.

Suggested Improvements

1.
Clarify Language Consistency and Tone
Ensure that phrasing across the document is consistent in level of formality and directness. For example:

- Instead of "This should include any errors produced by AI..." consider:

"Students should identify and discuss any errors in the AI-generated blogs..."

- Reword "There is no need to revise or polish these versions..." to:

"Do not edit or revise the original AI-generated outputs; we are interested in their unaltered form."

2.
Improve Formatting for Legibility
Consider presenting key instructions and expectations in bullet-point lists or tables to aid readability. This is particularly useful for:

- What to include in the Appendix.
- The components of the final blog and commentary.

3.
Clarify Terminology in Learning Outcomes

- "Editorial and reflective judgment" might be more intuitive as two separate outcomes:
    - Editorial judgment (focused on content refinement).
    - Reflective practice (focused on ethical and process insight).

Alternatively, explain in a footnote or introductory sentence what "editorial and reflective judgment" entails.

4.
Add Specific AI Tool Examples

- To support clarity and consistency, briefly specify that students should use free and accessible tools, such as:

Examples include Microsoft Copilot (GPT-4), Poe (Claude), or other browser-accessible LLMs.
This helps manage expectations and promotes equity of access.

5.
Enhance Reflection Section Criteria
Currently, the section on commentary/reflection is descriptive but could benefit from assessment descriptors (Pass/Merit/Distinction) similar to those used for the blog. This ensures parity in how both parts of the assessment are judged.
Add rubric language like:

- Distinction: Demonstrates deep insight into AI tool usage and revision choices, with critical and ethical reflection.
- Merit: Offers thoughtful commentary with some depth of analysis on AI outputs and revision process.
- Pass: Basic explanation of AI use and revision, with limited critical engagement.

6.
Reinforce Authenticity of Assessment
Add a note that blogs may be considered for publication on *The Mental Elf* platform (if appropriate). For example:
"This is an authentic assessment task, and selected blogs may be considered for publication on The Mental Elf platform, subject to editorial review."

7.
Add Links to Guidance Resources
Where applicable (e.g., CASP tools, blog writing resources, AI tools), embed hyperlinks:

- CASP Checklists
- APA 7 referencing guide (e.g., from KCL or OWL Purdue)
- King's AI Coversheet guidance (if publicly accessible)
